# Cloning and Functional Characterization of a Double-Stranded RNA-Degrading Nuclease in the Tawny Crazy Ant (*Nylanderia fulva*)

**DOI:** 10.3389/fphys.2022.833652

**Published:** 2022-01-28

**Authors:** Jiaxin Lei, Yongan Tan, Fabian List, Robert Puckett, Aaron M. Tarone, Edward L. Vargo, Keyan Zhu-Salzman

**Affiliations:** ^1^Department of Entomology, Texas A&M University, College Station, TX, United States; ^2^Institute of Plant Protection, Jiangsu Academy of Agricultural Sciences, Nanjing, China

**Keywords:** *Nylanderia fulva*, RNAi efficiency, dsRNA stability, dsRNase, midgut fluid

## Abstract

RNA interference is a powerful tool that post-transcriptionally silences target genes. However, silencing efficacy varies greatly among different insect species. Recently, we attempted to knock down some housekeeping genes in the tawny crazy ant (*Nylanderia fulva*), a relatively new invasive species in the southern United States, but only achieved relatively low silencing efficiency when dsRNA was orally administered. Here, we detected divalent cation-dependent, dsRNA-degrading activity in the midgut fluid of worker ants in *ex vivo* assays. To determine whether dsRNA degradation could contribute to low effectiveness of oral RNAi in *N. fulva*, we cloned its sole *dsRNase* gene (*NfdsRNase*). The deduced amino acid sequence contained a signal peptide and an endonuclease domain. Sequence alignment indicated a high degree of similarity with well-characterized dsRNases, particularly the six key residues at active sites. We also identified dsRNase homologs from five other ant species and found a tight phylogenetic relationship among ant dsRNases. *NfdsRNase* is expressed predominantly in the abdomen of worker ants. Oral delivery of dsRNA of *NfdsRNase* significantly reduced the expression of *NfdsRNase* transcripts, and substantially suppressed dsRNA-degrading activity of worker ants’ midgut fluids as well. Our data suggest that dsRNA stability in the alimentary tract is an important factor for gene silencing efficiency in *N. fulva*, and that blocking NfdsRNase in gut lumen could potentially improve RNAi, a novel pest management tactic in control of *N. fulva* and other ant species.

## Introduction

The tawny crazy ant (*Nylanderia fulva*), native to South America, is an invasive species that has rapidly spread in the southern United States. It has caused significant economic and ecological damage since it was first reported in Texas in 2002. It is a nuisance to homeowners and businesses because of its damage to electrical systems in urban areas (Mcdonald, 2012). *Nylanderia fulva* also has the potential to cause significant agricultural damage by tending and protecting honeydew-producing insect pests in the field (Meyers, 2008; Mcdonald, 2012). Ecologically, due to its high fecundity and ability to form supercolonies (Eyer et al., 2018), this ant species is extremely competitive and threatens biodiversity by displacing native ant species and non-ant arthropods (Lebrun et al., 2013). Due to the extreme density of workers and the spatially expansive nature of their supercolonies, effective control of this ant has been difficult. Therefore, development of an efficient and environmental-friendly approach to control *N. fulva* populations is urgently needed.

Gene silencing mediated by double-stranded RNAs (dsRNAs) suppresses gene expression post-transcriptionally in a sequence-specific manner (Baum and Roberts, 2014; Cooper et al., 2019). RNA interference (RNAi) technology is becoming one of the most promising tools for gene function studies, as well as a means of pest management in the field (Kim et al., 2015; Allen, 2021). However, RNAi efficiency varies greatly among different insect species, strains, developmental stages or tissue types (Baum and Roberts, 2014; Cooper et al., 2019). While coleopterans are very susceptible to ingested dsRNA, lower RNAi efficiency has been observed in lepidopterans and hemipterans (Baum and Roberts, 2014). Our previous work showed rather modest effects of RNAi via dsRNA feeding in *N. fulva* (Meng et al., 2020). Of all house-keeping genes we selected, none exceeded 40% reduction (Meng et al., 2020). Low potency is an obstacle in applying RNAi to management of *N. fulva* populations. Understanding the mechanisms underlying RNAi efficiency thus becomes imperative if RNAi is to be used as an effective control method.

Upon ingestion, dsRNA has to survive the hostile environment of the alimentary tract and enter cells in order to trigger the silencing machinery. Recent studies have demonstrated that degradation of dsRNA by dsRNA-degrading nucleases (dsRNase) may occur prior to cellular uptake. Extracellular degradation of dsRNA, notably in the gut, may explain the low efficiency of oral RNAi (Garbutt et al., 2013; Singh et al., 2017; Peng et al., 2018; Prentice et al., 2019; Cooper et al., 2020; Fan et al., 2020; Chen et al., 2021; Yoon et al., 2021). The first insect dsRNase identified in the midgut fluid of the domestic silk moth (*Bombyx mori*), contains a signal peptide and a non-specific endonuclease domain (Arimatsu et al., 2007). *Ex vivo* degradation assays showed that dsRNA stability can vary in the alimentary tracts of different insect species: dsRNA molecules persist longer in gut extracts of RNAi-sensitive red flour beetle (*Tribolium castaneum*) than in extracts from RNAi-insensitive pea aphid (*Acyrthosiphon pisum*) (Cao et al., 2018). Knockdown of *dsRNase* expression indeed greatly improves dsRNA stability in several insects across different orders, including the migratory locust (*Locusta migratoria*), the southern green stinkbug (*Nezara viridula*), the Queensland fruit fly (*Bactrocera tryoni*) and the Colorado potato beetle (*Leptinotarsa decemlineata*) (Song et al., 2017, 2019; Spit et al., 2017; Tayler et al., 2019; Sharma et al., 2021). Little research has been conducted to characterize dsRNases in ants.

We hypothesized that low RNAi efficacy in *N. fulva* was due to high dsRNase activity in the digestive tract, and that knockdown of its dsRNase enzymatic activity via gene silencing would significantly improve dsRNA stability. In this study, we attempted to detect dsRNA-degrading activity in worker ant midgut fluids. We cloned and characterized the sole *dsRNase* gene, *NfdsRNase*. In addition, we explored whether silencing *NfdsRNase* could prevent dsRNA from being degraded in the gut.

## Materials and Methods

### Insect Rearing

Tawny crazy ant (*N. fulva*) colonies were collected from College Station, Texas, United States and maintained in an environmentally controlled room (27°C, 80% RH) following procedures reported previously with minor modifications (Meng et al., 2020). Colonies were reared in clear 32-L plastic storage bins with a lid, the vertical sides of which were coated with Fluon to prevent ants from escaping. Harborages inside the container were Petri dishes (130 mm × 25 mm) with a layer (∼5 mm) of pre-wetted Castone^®^ plaster (Dentsply, York, PA, United States) at the bottom. Artificial diet (3.5% whey protein, 3.5% whole egg powder, 3.5% cricket powder, 16.5% sucrose, 0.1% vitamin mix, 0.1% methylparaben and 2% agar), dead cockroaches, as well as clear culture tubes (16 mm × 150 mm) half-filled with water or 13% sucrose, were provided to the colonies.

### Cloning the Full-Length *NfdsRNase* Gene

BmdsRNase (NP_001091744.1), a well-characterized dsRNase from *B. mori*, was used as the query sequence in a TBLASTN search^1^ against the *N. fulva* transcriptome database. To clone the full-length coding region of *NfdsRNase*, 50–60 worker ants were collected, frozen in liquid nitrogen and ground into fine power. Total RNA was extracted as described previously (Meng et al., 2020), and 2 μg was used to synthesize cDNA by M-MuLV reverse transcriptase (NEB, Ipswich, MA, United States) according to the manufacturer’s instructions with a gene-specific primer complementary to 3′ UTR of *NfdsRNase* (Table 1). The coding region was PCR-amplified using the above cDNA as templates and gene-specific primers (Table 1). The PCR product was purified by QIAquick^®^ Gel Extraction Kit (QIAGEN) and its sequence was confirmed by sequencing analysis. The mRNA sequence was used to search against the *N. fulva* genome via BLASTN to determine the copy number of *dsRNase*.

**TABLE 1:** Primers used in this study.

Purpose	Gene	Primer sequence (5′–3′)
cDNA cloning	*NfdsRNase*	Forward: ggggGAGCTCTATAATGCTGGTGCCGAATGTGC Reverse: ggggCTCGAGTTACATTAGAATGTCGATCACATCGAAC 3′ UTR: GACGGAATCAAGTGTTACTC
dsRNA synthesis	*NfdsRNase*	Forward: TAATACGACTCACTATAGGGAGAGGACGACTTCATTTACTTAGCA Reverse: TAATACGACTCACTATAGGGAGAGCGACATAATTCAATAGTGCGG
	*GFP*	Forward: TAATACGACTCACTATAGGGAGAGTTCTGCTGGTAGTGGTCGG Reverse: TAATACGACTCACTATAGGGAGAGACGACGGCAACTACAAGAC
RT-qPCR	*NfdsRNase*	Forward: GCTCGCCACAATTTCTCATCC Reverse: TCGTTCTTGAGCCAACTCGG

### Analysis of Deduced Amino Acid Sequences

*NfdsRNase* was used for transcriptome database searches to obtain mRNA sequences of other ant *dsRNase*s from *Camponotus floridanus*, *Solenopsis invicta*, *Atta cephalotes*, *Acromyrmex echinatior*, and *Linepithema humile* by BLASTN. Deduced amino acid sequences^2^ were used to obtain molecular masses and isoelectric points (pI) of dsRNases using PROTEIN CALCULATOR v3.4.^3^ SignalP-5.0 Server^4^ and SMART domain analysis^5^ were used to predict the existence of signal peptides and conserved functional domains, respectively. WoLF PSORT computational web tool^6^ was used to predict subcellular localization. Amino acid sequence alignment of all six ant dsRNases were performed with Clustal X2.1 software (Chenna et al., 2003), and then visualized by GeneDoc software (Nicholas et al., 1997). The NCBI Conserved Domain Search^7^ identified active site, including Mg^2+^-and substrate-binding sites in the DNA/RNA non-specific endonuclease domains predicted.

Phylogenetic relationships of full-length amino acid sequences were analyzed using Maximum Likelihood method by MEGA 7.0 and tested by the bootstrap method with 1,000 replicates. NCBI accession numbers of protein sequences used to construct the phylogenetic tree are shown in Table 2.

**TABLE 2:** NCBI accession numbers of amino acid sequences used to construct the phylogenetic tree.

Order	Species	Protein name	Accession number
Diptera	*Aedes aegypti*	AadsRNase	EAT42072.1
	*Anopheles darlingi*	AddsRNase1	ETN62076.1
		AddsRNase2	ETN61460.1
		AddsRNase3	ETN61459.1
	*Drosophila melanogaster*	DmdsRNase1	AAF49206.1
		DmdsRNase2	AAF49208.1
Coleoptera	*Diabrotica virgifera* virgifera	DvdsRNase1	QNH88357.1
		DvdsRNase2	QNH88358.1
		DvdsRNase3	QNH88359.1
	*Dendroctonus ponderosae*	DpdsRNase1	ENN82866.1
		DpdsRNase2	ERL84039.1
		DpdsRNase3	AEE63490.1
	*Tribolium castaneum*	TcdsRNase1	QJD55726.1
		TcdsRNase2	QJD55727.1
		TcdsRNase3	QJD55728.1
		TcdsRNase4	QJD55729.1
	*Leptinotarsa decemlineata*	LddsRNase1	APF31792.1
		LddsRNase2	APF31793.1
Hymenoptera	*Nylanderia fulva*	NfdsRNase	XP_029171190.1
	*Camponotus floridanus*	CfdsRNase	XP_011263277.1
	*Solenopsis invicta*	SidsRNase	XP_011156845.2
	*Linepithema humile*	LhdsRNase	XP_012230703.1
	*Acromyrmex echinatior*	AedsRNase	XP_011064189.1
	*Atta cephalotes*	AcdsRNase	XP_012064246.1
	*Vespula vulgaris*	VvdsRNase	KAF7408381.1
	*Vespa mandarinia*	VmdsRNase	XP_035717259.1
Orthoptera	*Locusta migratoria*	LmdsRNase1	ARW74134.1
		LmdsRNase2	ARW74135.1
		LmdsRNase3	ARW74136.1
		LmdsRNase4	ARW74137.1
	*Schistocerca gregaria*	SgdsRNase1	AHN55088.1
		SgdsRNase2	AHN55089.1
		SgdsRNase3	AHN55090.1
		SgdsRNase4	AHN55091.1
Lepidoptera	*Bombyx mori*	BmdsRNase1	XP_012547127.1
		BmdsRNase2	NP_001091744.1
	*Spodoptera littoralis*	SldsRNase	CAR92522.1
	*Spodoptera frugiperda*	SfdsRNase	CAR92521.1
Hemiptera	*Acyrthosiphon pisum*	ApdsRNase	XP_003242652.1
	*Diaphorina citri*	DcdsRNase1	XP_017297751.1
		DcdsRNase2	XP_008483858.1
	*Cimex lectularius*	CldsRNase1	XP_014241898.1
		CldsRNase2	XP_014241376.1
	*Frankliniella occidentalis*	FodsRNase	XP_026279236.1

### *In vitro* Synthesis of Double-Stranded RNAs

Double-stranded RNAs were synthesized using *in vitro* transcription with T7 RNA polymerase (NEB) following the manufacturer’s instructions with minor modification. The DNA templates for dsRNAs of *green fluorescent protein* (*GFP*) (ds*GFP*) and *NfdsRNase* (ds*NfdsRNase*) were PCR-amplified from pGL3-GFP and pET28a-NfdsRNase, respectively. T7 promoter sequences were added to 5′ ends of the primers (Table 1). PCR products were purified by QIAquick^®^ Gel Extraction Kit (QIAGEN). Each reaction of *in vitro* transcription contained 1 × RNAPol Reaction Buffer (NEB), 5 mM DTT (NEB), 0.5 mM rNTP (NEB), 1.25 U/μL T7 RNA polymerase (NEB), and 7.5 ng/μL DNA template. Reactions were incubated at 37°C overnight, and then treated by DNase I (NEB) and RNase One (Promega) simultaneously to remove DNA template and single-stranded RNA. dsRNA was purified by Ribozol™ reagent (VWR) following the manufacturer’s instructions. dsRNA concentrations were determined by Nanodrop.

### *Ex vivo* Assays of Double-Stranded RNA Stability in Worker Ant Midgut Fluid

Midguts were carefully dissected from worker ants under a dissecting microscope in pre-chilled 200 mM sodium acetate at pH 4.5, the physiological pH. The midgut fluids from 10 guts were collected in 10 μL of fresh dissection buffer. To do this, dissected guts were individually cut open with sharp forceps in the 10 μL buffer, into which gut fluid was released. For each sample, gut fluids from at least 50 midguts were pooled from at least five such collections. Pooled samples were centrifuged at 16,000 *g* at 4°C for 15 min. Supernatant was stored at −20°C in 10 μL aliquots until use. Protein concentration of the midgut fluids were determined by the Bradford assay (Bio-Rad, Hercules, CA, United States). Total proteins of 2 μg were used for degrading assays.

To evaluate dsRNA stability, 200 ng dsGFP was incubated with midgut fluids at 27°C for the time periods indicated, and then subjected to RNA extraction by RiboZol reagent (VWR) following the manufacturer’s instructions. The isolated samples were dissolved in 8 μL RNase-free water and examined on a 1.5% agarose gel by ethidium bromide (EtBr) staining. Band intensity was quantified by Photoshop and expressed as a ratio relative to the sample that was prepared the same way but was not incubated at 27°C.

### Feeding *NfdsRNase* Double-Stranded RNA to Worker Ants

Worker ants were starved for 6 h prior to RNAi treatment. Forty of them were transferred to a plastic container (20 cm × 15 cm × 10 cm) with a fine brush. The vertical sides of the plastic container were coated with Fluon. Harborage was made with a culture tube (13 mm × 100 mm) half-filled with water, which was plugged with a cotton ball and covered by aluminum foil. Water is also supplied by another culture tube without the aluminum foil cover. A wet cotton ball and three pieces of cardboard were placed in each container to retain moisture. For RNAi treatments, dsRNAs were administered orally via sucrose solution: treated *N. fulva* worker ants were fed on 13% sucrose solution containing 150 ng/μL ds*NfdsRNase* (30 μL in a PCR tube was put in each plastic container). The negative control consisted of ants fed on sucrose solution containing 150 ng/μL ds*GFP*. The dsRNA-containing sucrose solution, the only food source during the RNAi treatment, was replenished every 2 days. The ants were kept in the rearing room (27°C, 80% RH). After feeding on dsRNA-containing diet for 5 days, midgut fluids were collected as mentioned above for *ex vivo* assays of dsRNA stability and whole insects were harvested for gene expression analysis.

### Gene Expression Analysis of *NfdsRNase*

The expression of *NfdsRNase* was determined by quantitative RT-PCR (RT-qPCR). Heads, thoraxes, and abdomens were dissected from 50 to 80 *N. fulva* workers and instantly frozen in liquid nitrogen. For expression analysis of worker ants subjected to RNAi assays, entire bodies or abdomens of ∼40 workers were pooled in each replicate of each treatment and frozen in liquid nitrogen. All samples for gene expression analysis were stored at −80°C until further processing. Total RNA extraction and cDNA synthesis using random hexamer primers (Invitrogen, Carlsbad, CA, United States) were conducted as described above. Primers used in qPCR analysis were shown in Table 1. The *60S ribosomal protein L4* gene (*NfRPL4*) served as the internal control (Meng et al., 2020). qPCR reactions were run on a CFX384 Real-Time System (BioRad) using SYBR Green Mastermix reagent (BioRad). Relative fold changes of gene expression were calculated as we previously described (Zhu-Salzman et al., 2003).

### Statistical Analysis

All statistical analyses in this study were performed by SPSS software (v.20.0; SPSS Inc., Chicago, IL, United States). Relative gene expression data were analyzed by the independent samples’ Student’s *t* test.

## Results

### Midgut Fluids Exhibited Potent Double-Stranded RNA-Degrading Activities

**FIGURE 1:**
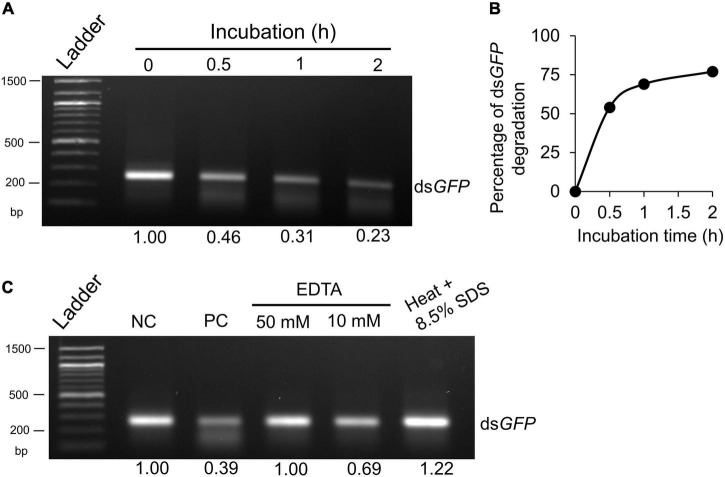
Degradation of double-stranded RNA (dsRNA) *ex vivo* by midgut fluids of worker ants. **(A)** ds*GFP* (200 ng) was incubated with 2 μg of midgut fluid proteins at 27°C for 0, 0.5, 1 and 2 h, respectively. **(B)** The percentage of ds*GFP* degradation in midgut fluids was calculated based on band intensity in panel **(A)**. **(C)** dsRNA degradation in midgut fluids was inhibited by EDTA or heat + SDS treatment. ds*GFP* (200 ng) was incubated for 0 (negative control, NC) or 2 h (positive control, PC) at 27°C with midgut fluids as above. ds*GFP* and midgut fluids were also incubated in the presence of EDTA (50 and 10 mM), or heated at 95°C for 30 min in 8.5% SDS. After incubation, samples were subjected to RNA extraction by Ribozol, separated on 1.5% agarose gel and visualized by EtBr staining under UV light. Band intensity was quantified by Photoshop and expressed as a ratio relative to the “0” or “negative control,” which was arbitrarily set at 1. Each experiment was repeated at least twice and a representative gel picture was presented.

To determine if the midgut lumen of worker ants possessed dsRNA-degrading activity, we assessed the stability of ds*GFP* in midgut fluid, followed by RNA extraction and agarose gel electrophoresis. The *ex vivo* assays showed that ds*GFP* was gradually degraded when incubated with midgut fluids, and only 23% remained after 2 h of incubation (Figures 1A,B). The degradation speed was highest during the first 0.5 h of incubation and slowly decreased afterward (Figure 1B). Moreover, EDTA, the chelating agent, effectively blocked degradation of ds*GFP* in gut fluids (Figure 1C), suggesting that the dsRNA degrading activity of the ribonuclease is metal ion-dependent. As expected, the denatured nuclease lost its enzymatic activity (Figure 1C). These data support that midgut fluids exhibit dsRNA-degrading activity, which in turn could contribute to relatively low RNAi efficacy in ants observed in our previous manuscript (Meng et al., 2020).

### Identification and Characterization of a *Nylanderia fulva* Double-Stranded RNA-Degrading Nucleases Gene

**FIGURE 2:**
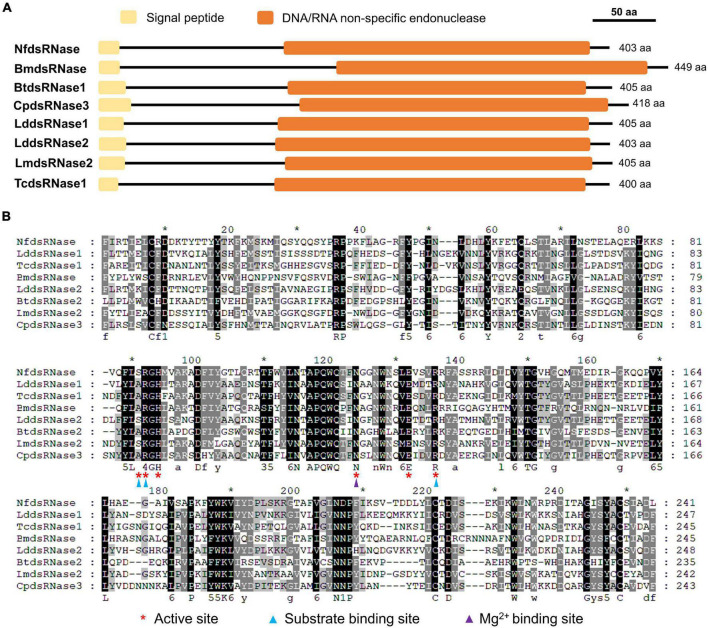
Analysis of deduced amino acid sequences of *NfdsRNases* with well-characterized dsRNases from other insects. **(A)** Schematic diagrams of key function domains of dsRNases. Yellow boxes indicate predicted signal peptides; Orange boxes denote the DNA/RNA non-specific endonuclease domains. **(B)** Multiple sequence alignment of the DNA/RNA non-specific endonuclease domains in insect dsRNases (Nf, *Nylanderia fulva*; Bm, *Bombyx mori*; Bt, *Bemisia tabaci*; Cp, *Cylas puncticollis*; Lm, *Locusta migratoria*; Ld, *Leptinotarsa decemlineata*; Tc, *Tribolium castaneum*). Red asterisks indicate the active sites. Substrate- and Mg^2+^-binding-sites are marked with blue and purple triangles, respectively. Numbers under the aligned sequences denote amino acid similarity groups of DN, EQ, ST, KR, FYW, and LIVM, respectively.

The presence of dsRNA-degrading activity in midgut fluid suggested the involvement of dsRNase. We searched the *N. fulva* transcriptome/genome and found only one *dsRNase* gene (*NfdsRNase*, XM_029315357). Multiple sequence alignment and functional domain analyses of NfdsRNase with well-characterized dsRNases from the domestic silk moth (*B. mori*), the African sweet potato weevil (*Cylas puncticollis*), the Colorado potato beetle (*L. decemlineata*), the red flour beetle (*T. castaneum*), the silver leaf whitefly (*Bemisia tabaci*), and the migratory locust (*L. migratoria*) (Liu et al., 2012; Luo et al., 2017; Song et al., 2017; Spit et al., 2017; Prentice et al., 2019; Peng et al., 2020) revealed that NfdsRNase contained key properties of a classic dsRNase. Similar to previously reported dsRNases, NfdsRNase contained a signal peptide (16 amino acids in length) and a highly conserved DNA/RNA non-specific endonuclease domain (Table 3 and Figures 2A,B). In the endonuclease domain of NfdsRNase, the six residuals at its putative active site were highly conserved, including substrate- and Mg^2+^-binding sites (Figure 2B). These molecular characteristics supported the extracellular nuclease nature of NfdsRNase.

**TABLE 3:** Characterization of *NfdsRNase* and seven previously reported insect dsRNases.

Name	Species	NCBI accession number	Number of amino acids (aa)	Molecular weight^1^ (kDa)	Isoelectric point^1^ (pI)	Signal peptide cleavage site^2^ (aa)	DNA/RNA non-specific endonuclease domain^3^ (aa) and similarity (%)^4^	Subcellular localization^5^
NfdsRNase	*Nylanderia fulva*	XP_029171190	403	46.0	9.03	16–17	146–386 (100%)	Extracellular
BmdsRNase	*Bombyx mori*	NP_001091744.1	449	50.9	9.35	16–17	188–432 (34.0%)	Extracellular
BtdsRNase2	*Bemisia tabaci*	XP_018913086.1	405	45.1	8.05	21–22	150–384 (31.9%)	Extracellular
CpdsRNase3	*Cylas puncticollis*	QCF41177.1	418	47.2	6.18	25–26	159–401 (32.9%)	Extracellular
LddsRNase1	*Leptinotarsa decemlineata*	APF31792.1	405	45.0	8.37	19–20	141–387 (34.0%)	Extracellular
LddsRNase2		APF31793.1	403	45.6	6.98	21–22	139–386 (36.3%)	Extracellular
LmdsRNase2	*Locusta migratoria*	ARW74135.1	405	43.8	4.98	21–22	147–388 (42.4%)	Extracellular
TcdsRNase1	*Tribolium castaneum*	QJD55726.1	400	44.4	4.97	15–16	139–383 (36.5%)	Extracellular

*^1^Molecular weight and isoelectric point was predicted based on the PROTEIN CALCULATOR v3.4 (http://protcalc.sourceforge.net/cgi-bin/protcalc).*

*^2^Signal peptide cleavage site was predicted according to the SignalP-5.0 Server (http://www.cbs.dtu.dk/services/SignalP/).*

*^3^Endonuclease_NS domain was predicted based on NCBI Conserved Domains (https://www.ncbi.nlm.nih.gov/Structure/cdd/wrpsb.cgi).*

*^4^Percentage of similarity to amino acid sequence of NfdsRNase in the DNA/RNA non-specific endonuclease domain was calculated by NCBI BLASTP suite-2sequences.*

*^5^Subcellular localization was predicted by the WoLF PSORT Prediction (https://www.genscript.com/wolf-psort.html).*

*bp, base pair; kDa, kilodalton; aa, amino acid.*

**FIGURE 3:**
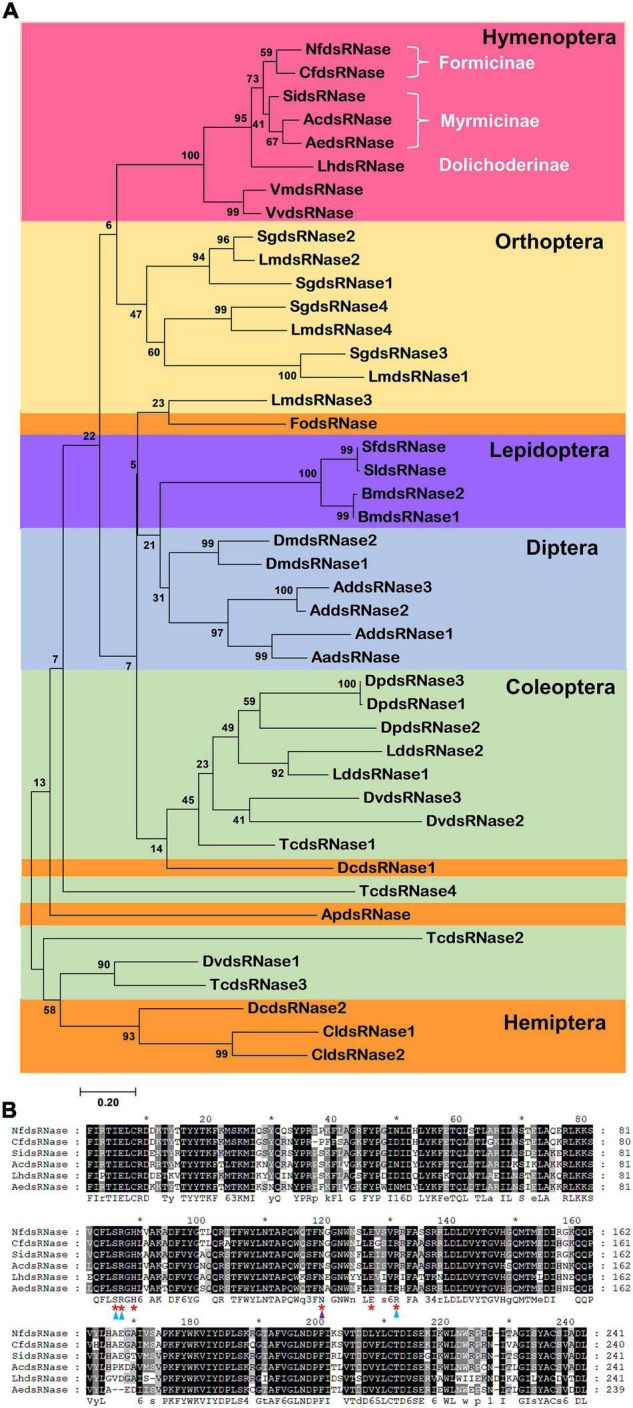
Phylogenetic analysis of insect dsRNases. **(A)** The phylogenetic tree of dsRNase proteins from insects belonging to six orders was constructed by MEGA 7.0 using Maximum Likelihood method. The confidence values (%) of each branch on the phylogenetic trees were calculated based on 1000 bootstrap replicates. Different shadings represent different insect orders. Coleoptera: Dp, *Dendroctonus ponderosae*; Dv, *Diabrotica virgifera virgifera*; Ld, *Leptinotarsa decemlineata*; Tc, *Tribolium castaneum*. Diptera: Aa, *Aedes aegypti*; Ad, *Anopheles darlingi*; Dm, *Drosophila melanogaster.* Hemiptera: Ap, *Acyrthosiphon pisum*; Cl, *Cimex lectularius*; Dc, *Diaphorina citri*; Fo, *Frankliniella occidentalis*. Hymenoptera: Ac, *Atta cephalotes*; Ae, *Acromyrmex echinatior*; Cf, *Camponotus floridanus*; Lh, *Linepithema humile*; Nf, *Nylanderia fulva*; Si, *Solenopsis invicta*.; Vv, *Vespula vulgaris*; Vm, *Vespa mandarinia*. Subfamilies are also marked. Lepidoptera: Bm, *Bombyx mori*; Sf, *Spodoptera frugiperda*; Sl, *Spodoptera littoralis*. Orthoptera: Lm, *Locusta migratoria*; Sg, *Schistocerca gregaria*. **(B)** Multiple sequence alignment of the DNA/RNA non-specific endonuclease domains in ant dsRNases. Red asterisks indicate the active sites. Substrate- and Mg^2+^-binding-sites are marked with blue and purple triangles, respectively.

Given the wide distribution of dsRNases in insects (Peng et al., 2020), it would be interesting to know their phylogenetic relationships. We performed phylogenetic analysis on 44 dsRNase protein sequences from 24 insect species from Coleoptera, Diptera, Hemiptera, Hymenoptera, Lepidoptera, and Orthoptera (Figure 3A). NfdsRNase was in the same clade with homologs from Hymenoptera, and consistent with their taxonomic relationship within ants (Ward, 2014). Notably, the six residues at the active site of the endonuclease domain were identical in all ants (Figure 3B). Homologs from Orthoptera were most closely related to those from Hymenoptera. Notably, dsRNases from Hemiptera appeared to be diverse.

### High Expression of *NfdsRNase* in the Abdomen of Worker Ants

**FIGURE 4:**
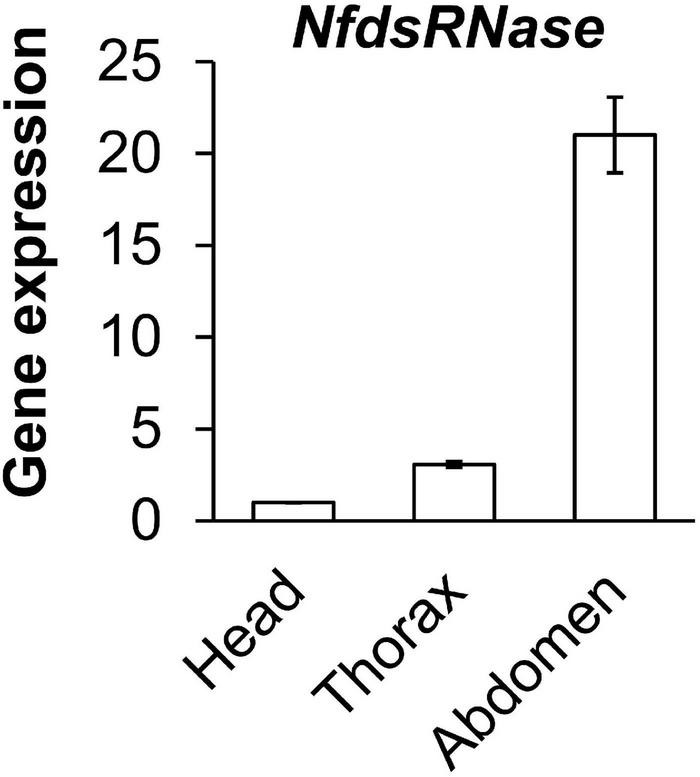
Relative expression of *NfdsRNase* in head, thorax and abdomen of worker ants. Gene expression was determined by RT-qPCR, with *60S ribosomal protein L4* served as the internal control.

We analyzed *NfdsRNase* expression in different segments of worker ants (head, thorax, and abdomen) by RT-qPCR (Figure 4). The highest expression was seen in abdomen, followed by thorax and head. The *NfdsRNase* transcript in abdomen was > 20-fold higher than that in the head, consistent with previous reports in other insect species that *dsRNase* is predominant expressed in insect guts (Peng et al., 2020; Chen et al., 2021). Enriched *NfdsRNase* transcripts in abdomen suggested that *NfdsRNase* was most likely present in the digestive tract of *N. fulva*.

### Knockdown of *NfdsRNase* Expression Significantly Reduced Double-Stranded RNA-Degrading Activity in Midgut Fluids

**FIGURE 5:**
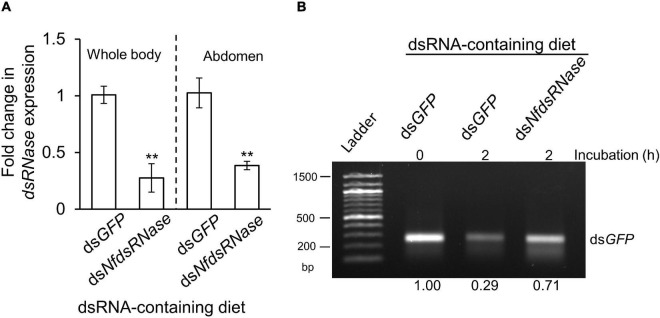
Contribution of *NfdsRNase* to dsRNA-degrading activity in worker ant midgut. After feeding worker ants with 13% sucrose solution containing 150 ng/μL ds*GFP* or ds*NfdsRNase* for 5 days, samples (whole body and abdomen) were harvested for gene expression analysis and midgut fluids were collected for *ex vivo* assays. Feeding on ds*NfdsRNase*-containing diet significantly reduced the expression of *NfdsRNase* **(A)** and dsRNase activity in midgut fluids **(B)** in worker ants. Gene expression analysis and *ex vivo* assays were performed as described above. Relative gene expression data were analyzed by the independent samples’ Student’s *t* test. Statistical significance is marked by **P < 0.01. dsRNAs incorporated into the 13% sucrose diet were shown above the gel image and duration of *ex vivo* incubation with ds*GFP* was also indicated.

To determine whether *NfdsRNase* was responsible for dsRNA degradation, we fed worker ants the dsRNA specific to *NfdsRNase* (ds*NfdsRNase*) incorporated into 13% sucrose solution. Workers fed on ds*GFP*-containing sucrose solution served as the negative control. Ingestion of ds*NfdsRNase* led to 72 and 61% transcript reduction in whole body and abdomen, respectively (Figure 5A). Incubation of ds*GFP* with midgut fluids collected from workers fed on diets containing ds*NfdsRNase* resulted in a notable 42% increase of dsRNA stability when compared to insects fed on ds*GFP* control (Figure 5B). Our results strongly supported that NfdsRNase was a key enzyme involved in dsRNA degradation in the midgut lumen.

## Discussion

RNAi-based control measures have great potential to be developed into environmental-friendly, species-specific insecticides (Whyard et al., 2009). Oral delivery of dsRNA is the most suitable for field applications due to feasibility and scalability (Peng et al., 2020; Allen, 2021). While this method has shown promise in some cases (Baum et al., 2007; Mao et al., 2011), it is less effective in triggering RNAi responses in other insects, such as *N. fulva* (Meng et al., 2020). Ingested dsRNAs have to undergo a number of physiological barriers prior to exerting their gene silencing action. Degradation by dsRNase in the digestive system is considered the first hurdle that dsRNAs come across (Cooper et al., 2019). In this study, we cloned and characterized the sole *NfdsRNase* gene from *N. fulva*. We not only showed its highest expression in abdomen, but also revealed the dsRNA-degrading activities of its protein product in midgut fluids of the worker ants. This is the first biochemical and genetic study of dsRNases in an ant species.

We initially proposed that degradation of dsRNA by NfdsRNase in the midgut lumen could be one of the limiting factors for RNAi efficiency through oral dsRNA delivery in *N. fulva*, and our data supported this hypothesis. Sequencing alignment revealed that NfdsRNase belonged to a superfamily of nucleases that degrade dsRNA (Friedhoff et al., 1994; Figures 2, 3). Key residues at the catalytic sites of the functional domain in NfdsRNase shared high similarity with previously characterized dsRNases (Liu et al., 2012; Song et al., 2017; Peng et al., 2020; Figure 2). In addition, the presence of the signal peptide (Figure 2 and Table 3) and high expression in the abdomen (Figure 3) indicated that NfdsRNase was likely an extracellular nuclease secreted into the midgut lumen. Moreover, inhibition of dsRNA-degrading activity by EDTA suggested that divalent cations play an important role in NfdsRNase-mediated nucleotide cleavage, consistent with other dsRNases (Cooper et al., 2020; Peng et al., 2020). Since silencing *NfdsRNase* resulted in a robust reduction of dsRNase activity (Figures 1, 5), degradation of dsRNA in midgut fluids was due largely to NfdsRNase in ants, which is also consistent with findings in other insects. Expression of functional recombinant NfdsRNase protein will allow us to learn additional properties, including substrate specificity, e.g., ssRNA, dsRNA, ssDNA, dsDNA, and circular plasmids.

Interestingly, there is only one copy of the *dsRNase* gene in all ant species we analyzed in this study, whereas most insect species possess at least two *dsRNase* genes. For example, *L. decemlineata* has two, *L. migratoria* has four, and the yellow fever mosquito (*Aedes aegypti*) have ten (Song et al., 2017; Spit et al., 2017; Giesbrecht et al., 2020). Multiple *dsRNase* genes are believed to be related to tissue-specific expression, functional divergence, and adaptation to various environments (Song et al., 2017, 2019; Giesbrecht et al., 2020). For instance, in *L. migratoria*, *LmdsRNase1* and *LmdsRNase4* are mainly expressed in hemolymph; while *LmdsRNase2* and *LmdsRNase3* are predominant in midgut. Among them, only LmdsRNase2 is a major contributor to dsRNA degradation (Song et al., 2017, 2019). It would be interesting to compare the enzymatic activity, substrate specificity, and environmental response between NfdsRNase and dsRNases from insects with multiple copies. In addition, ant dsRNases display remarkable sequence conservation with identical active site residues (Figure 3). Since variations in the first and second substrate binding sites have been long suggested to be related to differential substrate specificity (Wynant et al., 2014; Song et al., 2017, 2019), identical active site residues may imply that they share similar ribonuclease activities and thus the knowledge obtained from this research could be applicable to other ant species.

Recent studies have provided solid evidence that silencing *dsRNase* genes could significantly improve RNAi efficiency in a number of insects, including the African sweet potato weevil, the Colorado potato beetle, the red flour beetle, the silver leaf whitefly, the southern green stink bug, the migratory locust and the Queensland fruit fly (Luo et al., 2017; Song et al., 2017; Spit et al., 2017; Prentice et al., 2019; Tayler et al., 2019; Peng et al., 2020; Sharma et al., 2021). Therefore, knockdown of *NfdsRNase* should also lead to enhanced *N. fulva* RNAi efficacy. Additionally, alternative delivery systems-mediated by nanoparticles, such as chitosan, liposomes, and cationic dendrimers (Zhang et al., 2010; Zheng et al., 2019; Wang et al., 2020), should also be explored in future studies to assess their effectiveness to prevent dsRNA degradation and improve RNAi efficiency.

In summary, our NfdsRNase study is consistent with previously findings of several well-characterized dsRNase from other insects. Rapid degradation of dsRNA in midgut fluids is likely the key factor for modest RNAi effectiveness observed in our previous study (Meng et al., 2020). While it is important to enhance dsRNA stability in the digestive tract, other potential issues, such as limited cellular uptake, weak systemic transport, and insufficient RNAi core machinery need to be addressed (Cooper et al., 2019; Zhu and Palli, 2020) to develop effective RNAi strategies for control of *N. fulva* and other ant species.

## Data Availability Statement

The original contributions presented in the study are included in the article/supplementary material, further inquiries can be directed to the corresponding author.

## Author Contributions

JL, RP, AT, EV, and KZ-S designed the experiments. JL, YT, and FL performed the experiments. JL and KZ-S wrote the manuscript. All authors contributed to the article and approved the submitted version.

## Conflict of Interest

The authors declare that the research was conducted in the absence of any commercial or financial relationships that could be construed as a potential conflict of interest.

## Publisher’s Note

All claims expressed in this article are solely those of the authors and do not necessarily represent those of their affiliated organizations, or those of the publisher, the editors and the reviewers. Any product that may be evaluated in this article, or claim that may be made by its manufacturer, is not guaranteed or endorsed by the publisher.

## Footnotes

1
https://blast.ncbi.nlm.nih.gov/Blast.cgi

2
http://web.expasy.org/translate/

3
http://protcalc.sourceforge.net/cgi-bin/protcalc

4
http://www.cbs.dtu.dk/services/SignalP/

5
http://smart.embl-heidelberg.de/

6
https://www.genscript.com/wolf-psort.html

7
https://www.ncbi.nlm.nih.gov/Structure/cdd/wrpsb.cgi
